# Geochemical Fractionation and Environmental Risk Assessment of Potentially Toxic Elements in Copper Flotation Tailings from Tongling, Anhui Province

**DOI:** 10.3390/molecules31081349

**Published:** 2026-04-20

**Authors:** Yunhu Hu, Shuwen Xue, Mu You, Hongxia Fang

**Affiliations:** 1 Anhui Provincial Institute of Modern Coal Processing Technology, Anhui University of Science and Technology, Huainan 232001, China; huyunhu@ustc.edu.cn; 2 School of Chemistry and Materials Engineering, Huainan Normal University, Huainan 232001, China; hongxiafang@hnnu.edu.cn; 3 School of Earth and Space Sciences, University of Science and Technology of China, Hefei 230026, China; xueshuwen@cumt.edu.cn; 4 School of Biology Engineering, Huainan Normal University, Huainan 232001, China

**Keywords:** copper flotation tailings, potentially toxic elements, chemical fractions, ecological risk assessment

## Abstract

Copper flotation tailings are produced in large quantities during ore beneficiation and smelting, yet remain underutilized and can act as persistent sources of potentially toxic elements. Here, we combined XRD-based mineralogical characterization, ICP-OES quantification, Tessier sequential extraction, and pH-dependent batch leaching to elucidate metal occurrence, mobility, and associated ecological risk in tailings from Tongling, Anhui Province. This study systematically analyzed the mineral composition, potentially toxic elements content, chemical fractions, leaching behavior, and ecological risks of copper flotation tailings from the Shuimuchong tailings reservoir in Tongling, Anhui Province. XRD and XRF analyses revealed that calcite, quartz, and garnet were dominant mineral phases in the tailings. Elevated levels of Cu, Cd, Pb, Zn, and As were detected, some of which surpassed both local background concentrations and national soil quality standards. Most potentially toxic elements primarily existed in the residual fraction, indicating low mobility. Leaching experiments revealed that Zn, Cu, and As showed enhanced release under acidic conditions, making them priority risk elements during tailings acidification. Pollution index and ecological risk assessments indicated that the tailings were heavily contaminated, with Cu and Cd as the main risk contributors. The Risk Assessment Code (RAC) evaluation showed that Cd had the highest bioavailability and ecological risk. By clarifying the behavior of pollutants, this study contributes to the effective regulation of environmental hazards and the sustainable use of tailing materials.

## 1. Introduction

Mineral resources are essential to socioeconomic development, yet mining and mineral processing also generate large quantities of solid waste. Among these wastes, flotation tailings, which are fine-grained residues produced during ore beneficiation, constitute one of the major industrial solid wastes in China because of their large accumulation and relatively low utilization efficiency [1]. In particular, copper flotation tailings are generated in substantial amounts each year, creating long-term pressure on land occupation, environmental safety, and sustainable resource management [2].

Copper flotation tailings commonly contain elevated concentrations of potentially toxic elements (PTEs), including Cu, Pb, Zn, Cd, and As [3,4,5]. When such tailings are stored in the open air without effective control measures, rainfall infiltration, surface runoff, oxidation, and weathering can promote the release and migration of these elements into surrounding soils and water bodies, thereby causing secondary environmental contamination [6]. Once released into the environment, PTEs may accumulate in ecological receptors such as microorganisms, plants, and aquatic organisms and may subsequently enter the food chain, posing long-term risks to human health [7,8]. For this reason, the occurrence, migration, and risk of PTEs in mine tailings have attracted increasing attention.

Previous studies have shown that the environmental risk of PTEs is controlled not only by their total concentrations, but also by their occurrence forms and geochemical associations in environmental media [9,10]. In tailings systems, the mobility, bioavailability, and potential toxicity of PTEs are strongly influenced by mineral composition, physicochemical properties, and surrounding environmental conditions, especially pH and redox state [11]. Through dissolution, precipitation, complexation, adsorption, and mineral transformation, PTEs may occur in different geochemical fractions with distinct environmental behaviors [12]. Therefore, evaluation based solely on total elemental concentrations is insufficient for accurately assessing the environmental risk of tailings.

Sequential extraction methods have been widely used to assess the fractionation of metals in soils, sediments, and mine tailings because they provide operationally defined information on the partitioning of metals among different solid phases [13,14]. Among them, the Tessier sequential extraction procedure is one of the most commonly applied methods for distinguishing exchangeable, carbonate-bound, Fe–Mn oxide-bound, organically bound, and residual fractions [13,14]. This classification is useful for environmental assessment because different fractions generally correspond to different levels of mobility and environmental availability. In general, the exchangeable and carbonate-bound fractions are considered relatively more labile, whereas metals associated with the residual fraction are usually incorporated into mineral lattices and thus tend to be more stable under natural conditions [15]. Previous studies have reported that Cu, Pb, and Cr in mine tailings are often predominantly associated with relatively stable mineral phases such as chalcopyrite, galena, and chromite, although their actual environmental behavior remains highly dependent on site-specific mineralogical and geochemical conditions [16,17].

Although numerous studies have investigated heavy metal contamination in mining areas, many have focused mainly on total concentrations, pollution indices, or single aspects of environmental risk. Integrated investigations combining mineral composition, elemental concentrations, chemical fractionation, and ecological risk assessment remain comparatively limited for copper flotation tailings from Tongling. Tongling, a major copper-producing city along the Yangtze River Economic Belt, generates approximately five million tons of copper tailings annually, accounting for nearly one-third of its industrial solid waste [18]. The long-term accumulation of these tailings may result in sustained environmental pressure through the gradual release and transport of PTEs, thereby threatening surrounding ecosystems, agricultural safety, and human health.

In this context, a systematic investigation of the occurrence, fractionation, and ecological risk of PTEs in copper flotation tailings is necessary for understanding contaminant migration mechanisms and for supporting effective tailings management and pollution control. Therefore, this study selected a representative tailings pond in Tongling City to investigate the mineral composition, concentrations, chemical fractions, and ecological risk of environmentally sensitive elements. By integrating mineralogical characterization, elemental analysis, sequential extraction, and ecological risk evaluation, this work aims to clarify the occurrence and migration characteristics of PTEs in shallow sulfur-rich tailings and to provide a scientific basis for environmental risk prevention, tailings reutilization, and ecological restoration in mining-affected areas.

## 2. Materials and Methods

### 2.1. Sample Collection

**Figure 1:**
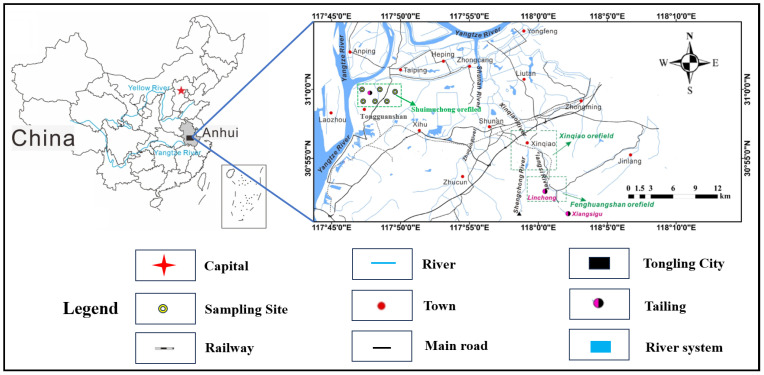
Location of the copper tailings area and distribution of sampling sites.

Flotation tailings samples were collected from the Shuimuchong tailings reservoir, located in Tongguanshan, Tongling City, Anhui Province (Figure 1). Although the Shuimuchong tailings reservoir is located within the catchment of a tributary of the Yangtze River, the objects and scale of this study are primarily confined to the tailings system itself, with the aim of elucidating the leaching behavior of metals and their environmental risk at the site scale; quantitative characterization of metal fluxes to adjacent river waters and the associated impacts on the aquatic environment is beyond the scope of this work. Set 2 sampling areas parallel to the direction of tailings discharge, as shown in Figure 1. Due to the discharge of gold ore dressing wastewater on the north side of the tailings pond, the content of some potentially toxic elements has increased abnormally. Therefore, sampling points were primarily located in an apparently unaffected area approximately 50 m outside the boundary of the impacted zone. At each sampling point, five subsamples of topsoil (0–20 cm) were collected within a 30 m radius using a Luoyang shovel–type hand auger (manual soil sampler), purchased from a commercial supplier in Luoyang, Henan, China.

The subsamples were placed on a clean plastic sheet in the field and homogenized with a plastic spatula to obtain a composite sample. Then, the quartering method was used for reduction, resulting in 1 representative soil sample. This study collected a total of 6 representative samples and numbered each sample sequentially, then transported to the laboratory. After air-drying, the samples were ground, passed through a 200-mesh sieve, and then sealed in glass containers for further analysis [19].

### 2.2. Sample Preparation and Analytical Methods

#### 2.2.1. Sequential Extraction Procedure

The fractionation profiles of the target elements were determined using the Tessier sequential extraction procedure, which is one of the most widely used operationally defined methods for evaluating metal partitioning in solid environmental matrices such as soils, sediments, and mine tailings. This method was adopted because, compared with total concentration analysis alone, it provides additional information on the distribution of metals among different solid phases and thus helps assess their potential mobility, environmental availability, and ecological risk under changing geochemical conditions. According to this procedure, the target elements were operationally partitioned into five fractions: exchangeable, carbonate-bound, Fe–Mn oxide-bound, organically bound, and residual (Table 1).

It should be noted that these fractions represent operationally defined associations rather than molecular-scale chemical species in the strict IUPAC sense. Therefore, the interpretation of the results in this study focused primarily on the relative mobility, potential release risk, and environmental significance of the different fractions rather than on direct identification of specific chemical species. In general, the exchangeable and carbonate-bound fractions are considered relatively more labile and environmentally available, whereas the residual fraction is usually regarded as more stable because metals in this fraction are commonly incorporated into mineral lattices.

For cross-validation, the fractionation behavior of Cu, Pb, and Zn was also evaluated using a modified Community Bureau of Reference (BCR) sequential extraction scheme. This method is also widely used in studies of contaminated soils and mine tailings and allows comparison of the obtained fractionation patterns with those reported in previous studies.

#### 2.2.2. Leaching Experiments

Prior to leaching, the samples were air-dried, homogenized, and sieved to <150 μm. Batch leaching tests were performed at a liquid-to-solid ratio of 5:1 (L kg^−1^) using solutions adjusted to initial pH values of 2.0, 4.0, 6.0, and 8.0. The suspensions were maintained at 25 °C under static conditions, with intermittent shaking to enhance solid–liquid interaction. Aliquots of the leachate on days 1, 4, 7, 10, 15, 24, and 30 under each pH condition were collected, filtered through 0.45 μm membranes, acidified with ultrapure HNO_3_, and stored at 4 °C prior to elemental analysis. The leaching mixtures were intermittently shaken every 24 h to enhance the interaction between the ash particles and the leaching solution. This allowed for a systematic evaluation of their leaching behavior and migration characteristics across different pH environments [20].

#### 2.2.3. Analytical Methods

Mineralogical phases of the tailings were identified by X-ray diffraction (XRD) using a Bruker D8 Advance diffractometer (Bruker AXS SE, Karlsruhe, Germany) with Cu Kα radiation (λ = 1.5406 Å) operated at 40 kV and 40 mA. The tailings were ground to a fine powder and pressed into pellets prior to analysis. Diffraction patterns were collected over a 2θ range of 5–70° at a scan rate of 4° min^−1^ with a step size of 0.02° and a counting time of 0.3 s per step. Phase identification was performed using JADE 6.0 with reference to the ICDD Powder Diffraction File (PDF) database. To ensure peak-position accuracy, the instrument zero shift/2θ offset was checked using the NIST SRM 640 silicon standard (National Institute of Standards and Technology, Gaithersburg, MD, USA). Major peak positions were verified prior to final phase assignment and cross-checked against published mineralogical reports for similar copper flotation tailings.

Major element composition of the tailings was determined by wavelength-dispersive X-ray fluorescence (WDXRF) using an ARL PERFORM’X 4200 spectrometer (Thermo Fisher Scientific, Ecublens, Switzerland). Quantification was conducted using the fundamental-parameter approach with matrix correction and spectral overlap treatment enabled. Instrument drift was monitored and corrected using a routine drift standard. Samples were oven-dried at 100 °C for 24 h, homogenized by grinding, and analyzed as pressed pellets. Calibration and result verification were performed using certified geological reference materials, including USGS BHVO-2 and BCR-2, to check both oxide quantification and drift correction performance. Analytical precision was assessed by replicate measurements (*n* = 3) and reported as relative standard deviation (RSD, %). Accuracy was evaluated using CRM recoveries, with 90–110% adopted as the acceptance criterion for major oxides; batches outside this range were recalibrated.

Tailings pH was measured using a pH meter (FE28-Standard, Mettler-Toledo GmbH, Greifensee, Switzerland) in a 1:2.5 (*w*/*v*) tailings-to-deionized water suspension. The suspension was intermittently stirred and equilibrated for 30 min, and pH was measured at 25 ± 1 °C. The pH meter was calibrated daily using standard buffer solutions at pH 4.00, 7.00, and 10.00. Each sample was measured in triplicate and reported as mean ± standard deviation.

For determination of pseudo-total concentrations of potentially toxic elements, 0.10 g of dried tailings was completely digested by microwave-assisted acid digestion following EPA Method 3052 using a mixed acid system of HF and HNO_3_. After digestion, the solution was diluted to 45 mL with ultrapure water and filtered through a 0.45 μm membrane. The filtration step was applied to remove residual particulates and to protect the ICP-OES sample introduction system. Therefore, the concentrations obtained in this study are reported as pseudo-total concentrations rather than absolute total concentrations in the strict geochemical sense. Prior to instrumental analysis, the digest solutions were appropriately diluted with ultrapure water, when necessary, to ensure that analyte concentrations fell within the corresponding calibration ranges. Concentrations of As, Cu, Cr, Ni, Cd, Pb, and Zn in the digests were determined by inductively coupled plasma optical emission spectrometry (ICP-OES; Avio 200, PerkinElmer, Shelton, CT, USA). Analytical wavelengths were As 188.979 nm, Cu 324.755 nm, Cr 267.716 nm, Ni 231.604 nm, Cd 226.502 nm, Pb 220.353 nm, and Zn 206.200 nm. Considering the concentration levels in the digests and the matrix characteristics of the samples, Cu and Zn were determined in radial view, whereas As, Cr, Ni, Cd, and Pb were determined in axial view. Spectral background was corrected using two-point off-peak background correction, with background positions set on both sides of each analytical line to account for continuum background and matrix-related baseline drift; when off-peak interference was observed, background positions were adjusted or the software auto-background routine was applied. Potential line overlaps were addressed using multicomponent spectral fitting to model baseline and neighboring interference contributions. If peak overlap or abnormal peak shapes persisted, alternative wavelengths with lower interference were selected. For element pairs with confirmed systematic overlap, inter-element correction was enabled to minimize spectral interference. External calibration was performed using at least five calibration points. The element-specific analytical parameters, including calibration ranges, plasma observation modes, linearity (R^2^), limits of detection (LOD), limits of quantification (LOQ), precision (RSD), and recovery, are summarized in Table S1. The pseudo-total concentrations obtained after digestion were used for contamination evaluation and ecological risk assessment, whereas the dissolved concentrations measured in the leachates were used to characterize the mobility and release behavior of metals under different pH conditions.

### 2.3. Results Analysis

#### 2.3.1. Geochemical Background Values and Pollution Indices

The geochemical background values used for pollution assessment were adopted from published regional soil background data for Anhui Province, China, which represent natural metal concentrations prior to significant anthropogenic disturbance. These background values were selected to ensure consistency and comparability with previous regional studies and commonly used environmental assessment practices. The regional background values for Cu, Pb, Cr, Zn, and As, together with the measured concentrations of the tailings, were used for pollution assessment. Pollution indices were calculated by comparing the measured concentrations of these elements in the copper flotation tailings with the corresponding background values.

#### 2.3.2. Single-Factor and Nemerow Pollution Indices

The degree of potentially toxic elements contamination in the study area was assessed using the single-pollution index (*P_i_*) and Nemerow comprehensive pollution index (P_N_) [21].

*P_i_* = *C_i_*/*C_i_*_0_ (1)

(2) $$P_{N}=\sqrt{\frac{\left(P_{\max}^{2}+{\bar{P}}^{2}\right)}{2}}$$

where the values of *P_i_* and *P_N_* were calculated using the following formulas: *C_i_* and *C_i_*_0_ represent the concentrations of potentially toxic elements *i* in the sample and the background value (mg/kg), respectively. *P_N_* was derived from the average and maximum values of *P_i_*. Pollution levels were categorized according to the criteria presented in Table 2.

#### 2.3.3. Potential Ecological Risk Index

The Potential Ecological Risk Index (PERI) method, proposed by Swedish scholar Hakanson, was used to assess potentially toxic elements pollution and its associated ecological risks [22]. Owing to its systematic and scientifically robust assessment of both contamination levels and associated ecological risks, this approach has been widely recognized and adopted as a standard method for evaluating potentially toxic elements pollution.

(3) $$E_{r}^{i}=T_{r}^{i}P_{i}$$

(4) $$RI=\sum E_{r}^{i}$$

The toxic response factor (*T_i_^r^*) represents a weighting coefficient assigned according to the relative toxicity of different potentially toxic elements. The *T_i_^r^* values for As, Cu, Cd, Cr, Zn, Ni, and Pb were 10, 5, 30, 10, 1, 5, and 5, respectively. Using these parameters, the individual potential ecological risk index (*E_i_^r^*) for each potentially toxic elements was calculated, and the sum of all *E_i_^r^* values constitutes the comprehensive potential ecological risk index (*RI*) [23]. The evaluation criteria were also shown in Table 2.

#### 2.3.4. Risk Assessment Code

The ecological risk associated with the bioavailability of potentially toxic elements was further assessed using the Risk Assessment Code (RAC), defined as follows:

(5) $$RAC=\frac{F_{1}+F_{2}}{C_{i}}*100\%$$

where *F*_1_ represents the content of the exchangeable fraction of potentially toxic elements *i*, and *F*_2_ represents the content of the carbonate-bound fraction of potentially toxic elements *i*. *C_i_* (mg/kg)denotes the pseudo-total concentration of potentially toxic element *i* determined after microwave-assisted acid digestion. The higher the proportion of active fractions of potentially toxic elements, the greater their potential environmental risk. Risk levels were classified into five categories: (1) RAC < 1%, no risk; (2) 1% < RAC ≤ 10%, low risk; (3) 10% < RAC ≤ 30%, moderate risk; (4) 30% < RAC ≤ 50%, high risk; and (5) RAC > 50%, extremely high risk [1].

### 2.4. Quality Assurance and Data Analysis

Strict quality assurance and quality control procedures were followed throughout sample preparation and analysis. Each analytical batch included reagent blanks and procedural blanks to monitor contamination, and the sequence for a set of six samples contained two blank samples, two control samples, and three duplicate samples as process controls. Each sample was measured in triplicate to ensure that the relative standard deviation of the results was below 5%. To further ensure data traceability and reproducibility, a unified QA/QC framework was established for XRD, WDXRF, ICP-OES, and, where applicable, the BCR sequential extraction.

For ICP-OES, external calibration was performed using at least five calibration points, with an acceptance criterion of R^2^ ≥ 0.999. Method precision was evaluated by duplicate digestion and duplicate measurement for at least 10% of the samples, and duplicate agreement was assessed using relative percent difference (RPD), with a typical acceptance criterion of RPD ≤ 20%. Analytical accuracy was verified using the certified solid-matrix reference materials GBW07305a and GBW07437, which covered the target elements As, Cu, Cr, Ni, Cd, Pb, and Zn. Element recoveries obtained from these reference materials ranged from 85% to 118%. Where necessary, matrix spikes and spike duplicates were additionally used to further assess method accuracy, and recoveries were generally maintained within 80–120%.

For WDXRF, quantification was conducted using the fundamental-parameter approach with matrix-effect correction and spectral-overlap treatment enabled. Calibration, result verification, and drift correction were carried out using the certified geological reference materials USGS BHVO-2 and BCR-2, together with a routine drift standard. The drift standard was measured at the beginning and end of each analytical batch, and control samples were inserted at a fixed frequency to monitor instrument stability. Analytical precision was assessed by replicate preparation and measurement, and accuracy was evaluated using CRM recoveries, with 90–110% adopted as the acceptance range for major oxides.

For XRD, a diffraction line-position standard was used to verify the 2θ zero point and peak-position consistency prior to sample measurements, and any required zero-shift correction was recorded. Phase identification was performed using JADE with the ICDD PDF database; key phase assignments were based on characteristic peak matching and peak-position consistency and were cross-checked against published mineralogical studies of similar copper flotation tailings. Statistical analyses were performed using Origin 2018 and SPSS 21.

The efficiency of the sequential extraction procedure was assessed by four replicate analyses of the BCR-701 certified sediment reference material. For each extraction step, percent recovery for each element was calculated as the ratio of the measured concentration to the certified value and is summarized in Table 3. The recovery ranges for the four fractions were 89–105% for F1, 94–108% for F2, 95–114% for F3, and 112–116% for Residual fraction. These results indicate that the method performance and analytical precision are acceptable, and the observed recoveries are consistent with ranges reported in the literature for complex sediment matrices [24,25].

## 3. Results and Discussion

### 3.1. Mineral Composition Analysis of Tailings

**Figure 2:**
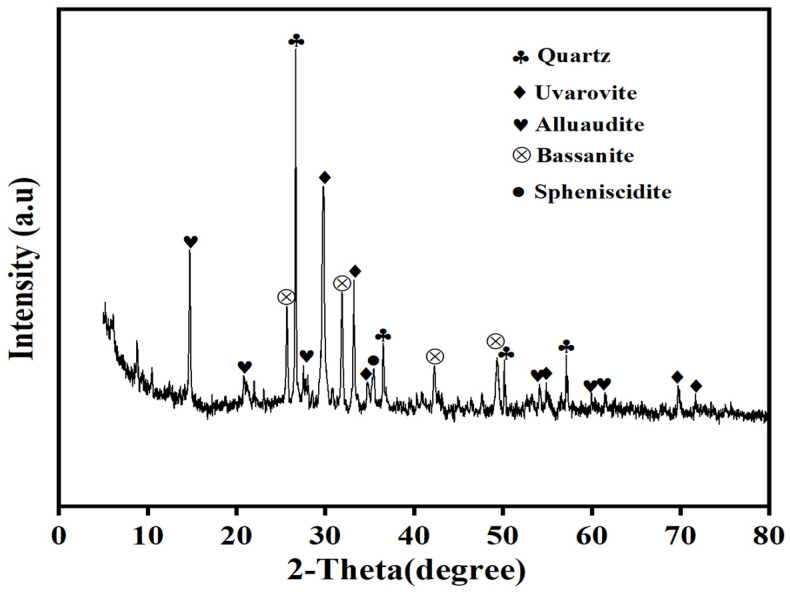
Mineral composition of copper tailings.

XRD analysis indicates that the copper flotation tailings are dominated by quartz, uvarovite, bassanite, and anuaudite, with minor amounts of spheniscidite (Figure 2). The bulk oxide composition determined by XRF is listed in Table 4, showing SiO_2_ (37.8%), CaO (27.3%), Fe_2_O_3_ (12.1%), and Al_2_O_3_ (9.21%) as the major components, with minor MgO (2.24%) and K_2_O (1.83%), and trace CuO (0.180%) and ZnO (0.0400%). LOI is 4.82%, indicating the presence of volatile components.

Previous studies reported broadly similar mineralogical assemblages for copper tailings in Tongling, suggesting limited spatial variability within the same metallogenic setting [26]. The Tongling copper deposits are typical skarn-type deposits within the Lower Yangtze metallogenic belt, where silicate gangue minerals together with carbonate components are common [27]. The XRF-derived bulk chemistry therefore provides complementary context consistent with a silicate–carbonate–sulfate matrix, but it should be noted that XRF does not identify mineral phases and is used here only as supporting chemical information. According to prior work on Tongling tailings, sulfide-bearing phases can undergo oxidative weathering, promoting sulfate accumulation and progressive acidification, which may enhance metal release under environmental exposure [28].

### 3.2. Content Characteristics of Trace Elements

The copper flotation tailings exhibit weak alkalinity (mean pH = 7.1 ± 0.48) and pronounced enrichment of several potentially toxic elements relative to local background values (Table 5). Compared with the local soil background values in Tongling [29], Cu, Cd, As, Zn, and Pb are enriched by factors of 34.4, 3.0, 7.0, 2.7, and 1.4, respectively. In addition, the concentrations of Cu and As exceed the Chinese soil risk screening values (GB 15618-2018) by approximately 11- and 3.34-fold, respectively.

The slightly alkaline pH may be associated with carbonate buffering (e.g., calcite dissolution), which neutralizes acidity generated during sulfide oxidation [30,31]. The pronounced enrichment of Cu and As suggests that the tailings can act as a long-term source of metal(loid)s to surrounding soils and waters, particularly under conditions that disrupt buffering capacity. Elevated concentrations in legacy tailings are also consistent with historical beneficiation practices and the retention of metal-bearing phases and reagents in the residual solids [32]. Compared with other similar copper tailings, it was found that the content of Cu and Zn in the tailings of this study was higher than that of other copper tailings [33,34]. For example, the content of Cu and Zn in the tailings of a copper mine in northern China is 122.10~338.00 mg/kg and 33.44~89.23 mg/kg, respectively. The content of Cu in the tailings of abandoned copper mines in Romania is 34.11~1048.17 mg/kg and 61.35~141.23 mg/kg, respectively. The higher Cu and Zn content in this study may be related to the frequent alternation of original ore composition, beneficiation process, and sand production direction [35].

**Table 5:** The concentrations of trace elements and pH values of the tailing (mg/kg) [36].

Items	Cd	Cr	Cu	Pb	Zn	As	Ni	pH
Min	0.22	22	8.8 × 10^2^	55	1.4 × 10^2^	63	4.7	6.8
Max	0.36	49	1.4 × 10^3^	92	3.4 × 10^2^	1.1 × 10^2^	15	7.5
Mean ± SD	0.30 ± 0.05	40 ± 8.8	1.1 × 10^3^ ± 1.9 × 10^2^	68 ± 12	2.3 × 10^2^ ± 64	84 ± 17	9.4 ± 3.2	7.1 ± 0.48
Background values of soils in Tongling	0.10	61	32	48	86	12	27	-
Soil quality standard (GB15618-2018) [37]	0.30	2.0 × 10^2^	1.0 × 10^2^	1.2 × 10^2^	2.5 × 10^2^	25	1.9 × 10^2^	-

Note: Chinese Soil Environmental Quality Standard for agricultural land (GB 15618-2018).

### 3.3. Fractionation of Trace Elements in Tailing

**Figure 3:**
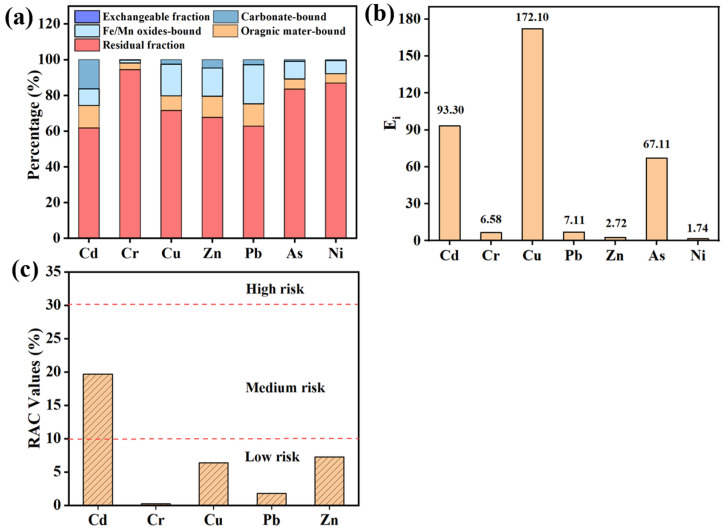
Percentage of trace elements in different fractions (**a**). The potential ecological risk assessment results for the trace elements (**b**). The RAC results for the trace elements (**c**). (Note: The potential ecological risk indices were calculated based on total metal concentrations and toxicity-response factors, without distinguishing specific oxidation states). Note: The blue color indicates a very low content and may not be clearly visible in the figure because of its extremely small proportion.

The mobility and bioavailability of potentially toxic elements were largely governed by their chemical fractionation, as different fractions exhibit distinct environmental behaviors and toxicities [38]. Most elements were predominantly associated with the residual fraction (F5), indicating generally low mobility under ambient conditions (Figure 3a; Table 6). The relatively higher labile fraction of Cd (F1) may be related to its weaker binding and greater tendency to associate with readily exchangeable and carbonate-related components; Cd can exhibit partial substitution behavior in Ca-bearing carbonate environments due to similar ionic radii [39]. In contrast, Cr shows the lowest proportion in the exchangeable fraction (F1, 0.131%) and the highest in the residual fraction (F5, 94.3%), suggesting its low mobility under natural conditions and a predominantly geogenic origin [40,41].

The Fe–Mn oxide-bound fraction (F3) of Cu, Zn, and Pb was relatively high, with average values of 17.6%, 15.7%, and 21.7%, respectively (Table 6), which is consistent with their strong affinity for Fe/Mn (hydr)oxides [42]. Apart from Cd, the F1 and F2 fractions of the other metals were generally low, which may be due to the surface sampling location. Prolonged weathering and leaching processes likely created acidic microenvironments that facilitated the loss of more mobile metal species from the surface layer [43]. Similar observations were reported by Zhang in the Hongtoushan tailings, where acid-extractable forms of Cr, Cd, Zn, Cu, and Pb in the top 0–15 cm layer were low due to decreased pH and active water movement [44].

In summary, Cd showed the highest environmental mobility, as reflected by its relatively larger proportion in labile fractions (F1–F2) and its higher release in leaching tests, followed by Zn and Cu. In contrast, Cr, Pb, As, and Ni primarily existed in stable residual forms (above 80%), indicating their geogenic origin, strong incorporation into silicate or sulfide mineral lattices, and relatively low ecological risk. This finding is consistent with previous studies on tailings mineralogy and potentially toxic elements fractionation [45].

### 3.4. Risk Assessment Based on Pollution Indices

The mean pseudo-total concentrations *(C_i_*) of trace metals in the tailings, obtained after microwave-assisted acid digestion, together with their corresponding background values (*C*_0_), were used to calculate the single pollution indices (*P_i_*), and the calculated P_i_ values are summarized in Table 7. According to the single-factor pollution index, all potentially toxic elements in the tailings except for Cr (0.660) and Ni (0.350) exceeded the pollution threshold. Based on the concentrations of potentially toxic elements in the flotation tailings, Pb (1.42) was classified as slightly polluted, Zn (2.72) as moderately polluted, while Cd (3.11), Cu (34.5), and As (6.71) were identified as heavily polluted. The Nemerow pollution index for surface tailings samples exceeded 3, indicating a high overall level of contamination in the tailings repository.

As shown in Figure 3b, the average potential ecological risk indices for Cd, Cr, Cu, Pb, Zn, As, and Ni were 93.30, 6.58, 172.10, 7.11, 2.72, 67.11, and 1.74, respectively. Among them, Cr, Pb, Zn, and Ni posed low ecological risks, whereas Cd and As posed high ecological risks. Although the concentration of Cd was relatively low, its risk level was elevated, likely due to its high toxicity response factor [46]. Cu exhibited a considerable ecological risk as well, attributed to its high concentration despite a moderate toxicity response factor (*T_i_^r^* = 5). The single-element ecological risk assessment indicated that Cd and Cu were the primary contributors to the potential ecological risk in the tailings. The mean comprehensive ecological risk index was 350.65, reflecting an overall high-risk level. Notably, Cd and Cu contributed 26.61% and 49.08% to the total RI, respectively, highlighting their roles as priority elements for risk mitigation and environmental management in the tailings area. Overall, Cd and Cu dominate the integrated ecological risk, indicating that mitigation efforts should prioritize these two elements.

Figure 3c presents the RAC values for seven potentially toxic elements in tailings to evaluate their potential ecological risks. Among them, Cd exhibits a significantly higher RAC value than the others, falling within the medium-risk range. This indicates that Cd possesses high environmental mobility and poses a considerable contamination threat, warranting priority in pollution control. In contrast, the RAC values of Cr, Cu, Pb, Zn, As, and Ni are all below 10%, placing them in the low-risk category. This suggests that these metals mainly exist in stable forms and pose relatively low immediate environmental risks. Overall, the ecological risks of potentially toxic elements in tailings vary considerably, underscoring the need for targeted risk management strategies based on RAC results.

### 3.5. Leaching Characteristics of Trace Elements

Given the elevated levels of potentially toxic elements in the Tongling copper tailings, batch leaching tests were conducted to evaluate pH-dependent release and potential mobility [47].

**Figure 4:**
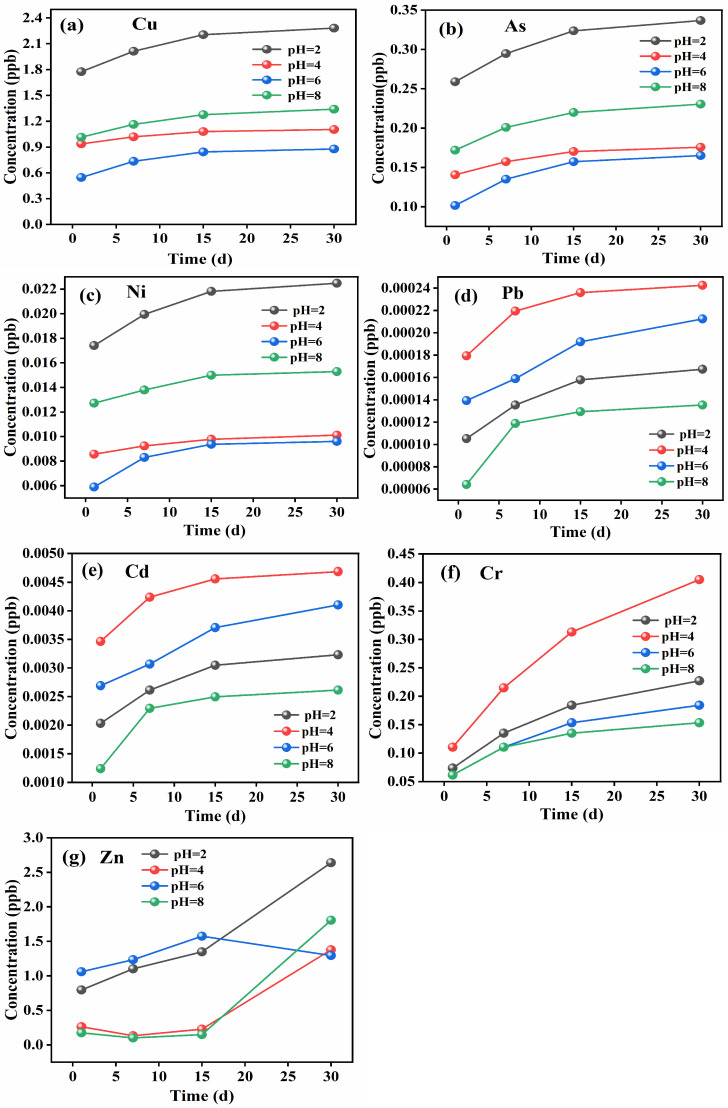
Leaching concentrations of trace elements in copper tailings leachate under different pH conditions: (**a**) Cu; (**b**) As; (**c**) Ni; (**d**) Pb; (**e**) Cd; (**f**) Cr; (**g**) Zn.

In Figure 4a, during the pH range of 4 to 6, the leached concentration of copper decreases as pH increases. The leaching amount of Cu reaches its maximum at pH = 2, which is 2.3 ppb. At pH = 6, Cu reaches the lowest leached concentration (~0.8 ppb). Most studies show that common copper-bearing compounds such as Cu_2_O, CuO, and Cu_2_S were basic or amphoteric in nature and exhibit higher solubility under acidic conditions. Consequently, they were particularly prone to dissolution in acidified leachates generated through sulfide oxidation and acid mine drainage processes [48]. However, the copper concentration in the leachate at pH = 8.0 was not the lowest, which may be attributed to the formation of complexes such as [CuCl_4_]^2−^ and [Cu(H_2_O)_4_]^2+^ between Cu^2+^, Cl^−1^, and H_2_O, enhancing copper solubility to some extent. These results suggest that pH interacts with mineralogical controls and solution chemistry (e.g., complexation), rather than acting as the sole governing factor [49].

As shown in Figure 4b, the leaching concentration of As increased over time under all pH conditions, indicating continuous release throughout the 30-day period. The highest leaching concentration was observed under strongly acidic conditions (pH = 2), reaching approximately 0.33 ppb by day 30. This suggests that acidification significantly enhances the mobility of As, likely related to the oxidative dissolution of As-bearing minerals phases and desorption under acidic condition. Under weakly alkaline conditions (pH = 8), the As concentration also remained relatively high (~0.23 ppb), possibly due to the formation of soluble arsenate complexes. In contrast, As release was lower under mildly acidic (pH = 4) and near-neutral (pH = 6) conditions, suggesting reduced solubility and potential adsorption onto iron or aluminum oxides. These results highlight the pH-sensitive nature of As release and underscore the environmental risks associated with acidification in tailings management [50,51].

Ni exhibited the highest release under strongly acidic conditions, followed by pH = 8.0, while leaching was minimal under mildly acidic to neutral environments. This pattern was likely due to the association of Ni with sulfide minerals of Cu and Zn [52]. A large amount of Ni was released on day one, with decreasing concentrations over time.

Compared to Cu, the leaching amount of Pb was significantly lower, which may be attributed to its chemical stability and low reactivity with dilute hydrochloric or sulfuric acid. As shown in Figure 4d, the highest Pb release occurred at pH = 4.0, followed by pH = 6.0, with the lowest release at pH = 8.0, indicating that Pb was more readily leached under moderately acidic conditions. Under strongly acidic conditions, an oxidation film may form on the surface of Pb-bearing minerals, inhibiting further dissolution and thus explaining the low release at pH = 2.0 [53].

Cd exhibited the most significant release under pH = 4.0 conditions, followed by pH = 6.0, while its release was relatively low under mildly acidic and alkaline conditions. As shown in Figure 4e, the leaching rate of Cd was extremely high during the initial stage, which may be attributed to the rapid oxidative decomposition of Cd-bearing sphalerite under acidic conditions [54].

Cr generally exhibits low solubility in many oxide/hydroxide forms; however, in this study its leaching shows a clear pH dependence. The highest Cr concentration in the leachate occurred at pH 4, reaching ~0.40 ppb after 30 days (Figure 4f), whereas lower concentrations were observed under strongly acidic conditions (pH 2) and near-neutral to alkaline conditions (pH 6–8). This behavior contrasts with that of several other elements that display enhanced release under strongly acidic conditions, suggesting that moderately acidic environments may be more favorable for Cr mobilization from the tailings matrix, potentially due to pH-controlled dissolution/desorption processes of Cr-bearing phases. Overall, the results indicate that Cr release is sensitive to pH, with pH ≈ 4 representing a condition of comparatively higher Cr leachability [55].

As shown in Figure 4g, Zn exhibited markedly different leaching behaviors under varying pH conditions. Under strongly acidic conditions (pH = 2), the release of Zn was significantly higher than that under other pH values, reaching a maximum concentration of approximately 2.5 ppb on day 30, indicating that acidic environments favor Zn dissolution and migration. Under alkaline conditions (pH = 8), Zn concentrations in the leachate were relatively low during the first 15 days but increased rapidly afterward, eventually reaching around 1.8 ppb. This may be attributed to the amphoteric nature of Zn and the formation of soluble hydroxyl complexes. Overall, Zn shows high release potential under both strongly acidic and mildly alkaline conditions, suggesting that its mobility should be closely monitored during tailings acidification and alkalization processes.

The leaching behavior of different potentially toxic elements in tailings is highly sensitive to pH conditions. Acidic environments significantly enhance the release of Cd, Zn, and As, while Cr shows peak mobility under moderately acidic conditions. Pb and Ni exhibit lower leachability but still pose potential risks. These findings highlight the need for tailored remediation strategies based on site-specific pH conditions.

## 4. Conclusions

This study systematically analyzed the mineral composition, potentially toxic elements content, chemical fractions, leaching behavior, and ecological risks of copper tailings from the Shuimuchong tailings reservoir in Tongling, Anhui Province, China. XRD and XRF analyses showed that the tailings mainly consist of stable silicate, carbonate, and phosphate minerals, explaining the dominance of Cr, Pb, Ni, and As in the residual fraction with low mobility and bioavailability. In contrast, Cu, Zn, and Cd were partly associated with carbonate- and Fe/Mn oxide-bound phases, showing higher leaching potential under acidic conditions. These results suggest that the mineralogical matrix exerts an important control on the fractionation and potential mobility of metal(loid)s. In addition, Cu, Cd, As, Zn, and Pb are significantly enriched in the tailings relative to local soil background values, and Cu and As exceed the Chinese soil risk screening values (GB 15618-2018). Fractionation analysis indicated that most potentially toxic elements predominantly exist in the residual fraction. Leaching experiments under different pH conditions demonstrated that Zn, Cu, and As exhibit higher release potential in acidic environments, identifying them as key risk elements during tailings acidification. Ecological risk assessments based on multiple pollution indices identified Cu and Cd as the primary contributors. Both the single-factor index and Nemerow comprehensive index indicated severe contamination, and the PERI classified the area as high risk, with Cd and Cu contributing 26.61% and 49.08% of the total ecological risk, respectively. The RAC further highlighted Cd as having the highest bioavailability and ecological hazard. In summary, this study elucidates the geochemical behavior and potential environmental risks of potentially toxic elements in copper tailings, providing a scientific basis for environmental risk management, pollution control, and resource utilization in mining areas.

## Figures and Tables

**Table 1:** Tessier sequential extraction procedures for metal fractionation analysis.

Sequences	Fractionations	Extractants	Extractant Conditions
1	Exchangeable fraction (F1)	8 mL 1 mol·L^−1^ MgCl_2_ solution, pH = 7	25 °C oscillation for 1 h
2	Carbonate-bound fraction (F2)	8 mL 1 mol·L^−1^ NaAc solution, pH = 5	25 °C oscillation for 5 h
3	Organic matter-bound fraction (F3)	40 mL 0.04 mol·L^−1^ NH_2_OH·HCl (25% HAc)	95 °C oscillation for 6 h
4	Fe/Mn oxides-bound fraction (F4)	3 mL 0.01 mol/L HNO_3_ + 5 mL 30% H_2_O_2_ + 5 mL 3.2 mol·L^−1^ NH_4_Ac (20% HNO_3_) solution	85 °C oscillation for 2 h, twice. 25 °C oscillation for 0.5 h
5	Residual bound (F5)	HF + HCl + HClO_4_ + HNO_3_	Dissolved until the solution is clear and transparent

**Table 2:** Grading standards of environmental quality and potential ecological risk.

Environmental Quality			Potential Ecological Risk		
Single Factor Index and Nemerow Index	Degree	Pollution Level	$E_{r}^{i}$	*RI*	Risk Level
*P_i_*, *P_N_* ≤ 0.7	0	Uncontaminated	<30	<100	Slight
0.7 < *P_i_*, *P_N_* ≤ 1.0	I	Alert	30 ≤ $E_{r}^{i}$ < 60	100 ≤ *RI* < 200	Medium
1.0 < *P_i_*, *P_N_* ≤ 2.0	II	Slightly	60 ≤ $E_{r}^{i}$ < 120	200 ≤ *RI* < 400	Strong
2.0 < *Pi*, *P_N_* ≤ 3.0	III	Moderate	120 ≤ $E_{r}^{i}$ < 240	≥400	Very strong
*Pi*, *P_N_* > 3.0	IV	High	≥240		Extremely strong

**Table 3:** Recovery (%) of reference material BCR-701(mean ± sta ndard deviation, *n* = 4).

Metals	Fraction 1	Fraction 2	Fraction 3	Residual Fraction
Cd	98 ± 2	105 ± 2	110 ± 4	112 ± 4
Cr	102 ± 2	98 ± 3	102 ± 2	115 ± 2
Cu	105 ± 4	100 ± 2	95 ± 2	116 ± 2
Zn	94 ± 1	94 ± 1	103 ± 2	115 ± 2
Pb	89 ± 2	102 ± 2	106 ± 2	114 ± 5
Ni	102 ± 2	108 ± 2	101 ± 2	112 ± 3

**Table 4:** Results of XRF Spectrum Analysis.

Chemical Compositions	Percentage (%)
SiO_2_	37.8
CaO	27.3
Fe_2_O_3_	12.1
Al_2_O_3_	9.21
MgO	2.24
K_2_O	1.83
CuO	0.180
TiO_2_	0.420
ZnO	0.0400
Na_2_O	0.520
MoO_3_	3.54
Loss on ignition	4.82

**Table 6:** Percentage distribution of trace metals in different chemical fractions (F1–F5) in the tailings (%).

Metals	F1	F2	F3	F4	F5
Cd	0.824	16.2	9.19	12.5	61.3
Cr	0.131	0.171	1.71	3.67	94.3
Cu	0.483	2.52	17.6	8.17	71.2
Zn	0.852	4.62	15.7	11.7	67.1
Pb	0.672	2.81	21.7	12.4	62.4
As	0.752	0.891	9.79	5.68	82.9
Ni	0.263	0.482	7.33	5.21	86.7

**Table 7:** Single pollution indices (*P_i_*) of trace metals in copper tailings.

Items	Cd	Cr	Cu	Pb	Zn	As	Ni
*C_i_*	0.300	40.1	1.11 × 10^3^	68.0	233	84.0	9.40
*C_i_*_0_	0.100	61.0	32.2	47.8	85.6	12.4	26.9
*P_i_*	3.11	0.660	34.5	1.42	2.72	6.71	0.350

## Data Availability

The datasets used and/or analyzed during the current study available from the corresponding author on reasonable request.

## Supplementary Materials

The following supporting information can be downloaded at: https://www.mdpi.com/article/10.3390/molecules31081349/s1, Table S1. Element-specific analytical parameters and validation results for ICP-OES determination of total digests of copper tailings.
