# Supplementary material for: Geochemical Fractionation and Environmental Risk Assessment of Potentially Toxic Elements in Copper Flotation Tailings from Tongling, Anhui Province
Source: Molecules. 2026 Apr 20;31(8):1349. doi: 10.3390/molecules31081349 (PMC13118756; doi:10.3390/molecules31081349)
Supplement: Supplementary file 1 [file molecules-31-01349-s001.zip › molecules-4206682-supplementary.pdf]

Supporting Information

Table S1. Element-specific analytical parameters and validation results for ICP-OES determination of total digests of copper tailings

Elements	Wavelength (nm)	Plasma view	Calibration range (μg/L)	R^2	LOD （ug/L)	LOQ （ug/L)	Precision (RSD, %)	Recovery (%)
As	188.979	axial	0,10,20,50,100,200	0.9993	0.10	0.30	3.6	95
Cu	324.755	radial	0,10,20,50,100,300	0.9991	0.03	0.10	2.8	98
Cr	267.716	axial	0,10,20,50,100,200	0.9995	0.08	0.25	1.9	96
Ni	231.604	axial	0,5,10,20,50,100	0.9998	0.05	0.15	2.7	94
Cd	226.502	axial	0, 0.1, 0.2, 0.5, 1, 5	0.9997	0.01	0.03	3.4	93
Pb	220.353	axial	0,10,20,50,100,200	0.9992	0.03	0.10	4.2	95
Zn	206.200	radial	0,10,20,50,100,200	0.9994	0.02	0.06	1.5	98

Note: Digest solutions were appropriately diluted with ultrapure water prior to ICP-OES determination, when necessary, to ensure that analyte concentrations fell within the corresponding calibration ranges. The LOD and LOQ values were determined from repeated measurements of low-level standards and/or blanks. Precision was expressed as relative standard deviation (RSD) based on replicate digestion and measurement, whereas accuracy was evaluated using certified reference materials and recovery tests.
